# An Electronic Medication Module to Improve Health Literacy in Patients With Type 2 Diabetes Mellitus: Pilot Randomized Controlled Trial

**DOI:** 10.2196/13746

**Published:** 2020-04-28

**Authors:** Hanna Marita Seidling, Cornelia Mahler, Beate Strauß, Aline Weis, Marion Stützle, Johannes Krisam, INFOPAT P4/P5 Study Team, Joachim Szecsenyi, Walter Emil Haefeli

**Affiliations:** 1 Department of Clinical Pharmacology and Pharmacoepidemiology Heidelberg University Hospital Heidelberg Germany; 2 Cooperation Unit Clinical Pharmacy Heidelberg University Hospital Heidelberg Germany; 3 Department of General Practice and Health Services Research Heidelberg University Hospital Heidelberg Germany; 4 Department of Nursing Science University Hospital Tuebingen Tuebingen Germany; 5 Institute of Medical Biometry and Informatics Heidelberg University Hospital Heidelberg Germany; 6 See Acknowledgments

**Keywords:** medication self-management, patient empowerment, health literacy, chronic diseases, type 2 diabetes mellitus, electronic health record, PEPA, electronic medication module, structured medication review

## Abstract

**Background:**

In primary care, patients play a crucial role in managing care processes and handling drug treatment. A decisive factor for success is their health literacy, and several interventions have been introduced to support patients in fulfilling their responsibility.

**Objective:**

The aim of this study is to assess the influence of such an intervention (ie, a medication module) within a patient-led electronic health record on patients’ health literacy.

**Methods:**

We conducted a randomized controlled study among community-dwelling patients with type 2 diabetes mellitus. Patients were recruited from primary care practices. After randomization, patients either had access to an internet-based medication module allowing them to store their medication information, look up drug information, and print a medication schedule (intervention group), or they received an information brochure on the importance of medication schedules (control group). After 4-8 weeks, all patients were invited to attend a structured medication review (ie, follow-up visit). Data were collected via questionnaires before the start of the intervention and during the follow-up visit. The main outcome measure was the mean difference in health literacy between baseline and follow-up assessments of patients in the control and intervention groups.

**Results:**

Of 116 recruited patients, 107 (92.2%) completed the follow-up assessment and were eligible for intention-to-treat analyses. Only 73 patients, of which 29 were in the intervention group, followed the study protocol and were eligible for per-protocol analysis. No differences in overall health literacy were observed in either the intention-to-treat or in the per-protocol cohorts. Reasons for a null effect might be that the cohort was not particularly enriched with participants with low health literacy, thus precluding measurable improvement (ie, ceiling effect). Moreover, the success of implementation was considered poor because both the correct application of the study procedure (ie, randomization according to the protocol and dropout of 29 patients) and the actual interaction with the medication module was modest (ie, dropout of 9 patients).

**Conclusions:**

The conduct of this randomized controlled study was challenging, leaving it open whether inadequate implementation, too short of a duration, or insufficient efficacy of the intervention, as such, contributed to the null effect of this study. This clearly outlines the value of piloting complex interventions and the accompanying process evaluations.

## Introduction

In the course of their disease, patients with type 2 diabetes mellitus (T2DM) depend on the lifelong intake of drugs, which requires continuous unremitting efforts to self-manage the medication process. Thereby, medication self-management refers not only to active drug administration but also to filling and picking up prescriptions, understanding the medication regimen, integrating it in a daily schedule, monitoring drug effects, and, finally, sustaining it over the long term [1].

Besides the complexity of the disease and the medication regimen, medication self-management capacity is also influenced by patient characteristics, such as patients’ health literacy [1,2]. According to Sørensen and coworkers, health literacy “... entails people’s knowledge, motivation, and competencies to access, understand, appraise, and apply health information in order to make judgments and take decisions in everyday life concerning healthcare, disease prevention, and health promotion to maintain or improve quality of life during the life course” [3]. All four dimensions of health literacy are relevant within medication self-management: (1) access and obtain information relevant to health, (2) understand information relevant to health, (3) process and appraise information relevant to health, and (4) apply and use information relevant to health. Health literacy, therefore, plays an important role in medication self-management because limited health literacy can be a barrier to medication reconciliation [3] and can be associated with medication beliefs [4,5] and medication nonadherence [4,6].

Improving health literacy has been targeted by a number of single and mixed interventions aimed at improving comprehension [7] or provision of programs supporting patients to actively fulfill their roles in medication self-management as do patient portals, for instance [8]. Patient portals are often interlinked with electronic health records (EHRs), which, until today, have the predominant aim to store all disease-related documentation, facilitating interchange and transfer of information between health care professionals. Current evidence on patients’ attitudes toward patient portals suggest that patients might not use these portals on a regular basis [9], for instance, because they are not familiar with the functionalities they offer [10]. Even if the functionalities are known, the handling might still be complicated for the target population or they might be reluctant to use them, as has been shown for additional functionalities, such as secure messaging. Hence, these functionalities should always be assessed for potential barriers in usage [11]. However, if patients are individually informed and trained according to their needs on how to use the portal, and a particular effort is put on system usability from the beginning, patient portals might be useful tools to strengthen the patient’s role [10,12,13].

In 2012, a patient-controlled PEPA (personal EHR) was launched in the region of Rhine-Neckar in the southwest of Germany, within the INFOPAT (INFOrmation Technology for PATient-oriented Healthcare in the Rhine-Neckar metropolitan region) project [14]. The PEPA is “owned” by the patient so that he or she gains control over all disease-associated and medication-related data shared in the PEPA [15,16]. The PEPA offers the function of documentation but also actively involves patients in his or her process of care. In contrast to previous EHRs, patients can customize their PEPA themselves and give health care professionals access to its content [17]. To further support patients, the PEPA was equipped with an interactive medication module where patients can access information on different drugs, check drug-drug interactions, get evidence-based information on diabetes and its drug treatment, and compose and update their personal medication schedule in order to support medication self-management.

In total, the PEPA with the medication module forms a complex intervention that might support patients in fulfilling their active roles in the medication process and during drug handling. To assess the medication module’s effects, a complex and well-piloted study design will be required. Thereby, it will be particularly challenging to analyze causal relationships for success or reasons for failure of such an intervention, which may both result from inadequate implementation as well as inadequate effects.

The aim of this study was to pilot the feasibility of the study procedure (ie, patient recruitment, randomization, data assessment, and documentation in the primary care practices) and of the intervention (ie, conduction of training episodes and accessibility of the medication module), while at the same time assessing the influence of the medication module on health literacy.

## Methods

### Overview

We performed an unblinded, exploratory, prospective, randomized controlled study with two data collection points among community-dwelling patients attending primary care practices. The study protocol was approved by the Ethics Committees of the Medical Faculty of Heidelberg University (S-540/2015) and of the State Medical Board of the state of Baden-Württemberg, Germany.

### Recruitment

Primary care practices were recruited via a practice network of the Department of General Practice and Health Services Research at Heidelberg University Hospital, Germany. In each practice, the responsible general practitioner (GP) agreed to participate and entrusted a medical assistant with the coordination of the study in his or her practice and the conduct of a structured medication review with all included patients. Because the medical assistants were asked to evaluate each structured medication review, they were also included as study participants and signed informed consent forms.

Patients were eligible if they were 18 years of age or older, were diagnosed with T2DM and treated with oral antidiabetic drugs and/or insulin, were considered mentally and physically capable of participating in the study, were generally familiar with computer use, had access to a computer or mobile device with internet access and an email account, and consented in written form to participate in the study. Patients not fulfilling the inclusion criteria could not participate in the study; apart from that, there were no formal exclusion criteria.

Each participating primary care practice received €100 for study-related expenditures, all participating medical assistants received compensation of €200 plus €40 per patient, and patients received compensation of €50 for their efforts.

### Intervention

The intervention comprised the personal use of the medication module that was embedded in the PEPA. The medication module was developed based on previous qualitative focus group discussions with T2DM patients, GPs, and pharmacists, who defined user requirements [18]. The user requirements were transferred to an initial prototype of the medication module that was then pretested with 6 patients and subsequently optimized considering the patients’ feedback. Finally, the module consisted of a documentation submodule for the patients’ medication regimen and a general information portal on drug administration. The documentation submodule facilitated searching all drugs available on the German drug market, putting together a medication regimen, and entering additional information, such as drug dosage, indication, and administration advice. Subsequently, the patients could store the medication regimen to access and modify in the future. Moreover, they could print out a medication schedule containing all previously entered information. For each drug, the patient had access to basic drug information, such as the package information leaflet and more detailed information on drug administration (eg, on whether a tablet could be split or crushed). Moreover, the entire medication regimen was checked for drug-drug interactions involving over-the-counter drugs as well as contraindicated combinations.

Before using the medication module for the first time, each patient completed a 45-minute, face-to-face, hands-on structured training session by a member of the study team and printed education material on how to use the module. Moreover, patients could approach a first-level support person in case they had problems using the medication module of the PEPA. However, participants were not contacted proactively to ensure that they used the tool.

Participants of the control group received a brochure about the importance and content of a medication schedule.

### Study Procedure and Outcome Measures

**Figure 1:**
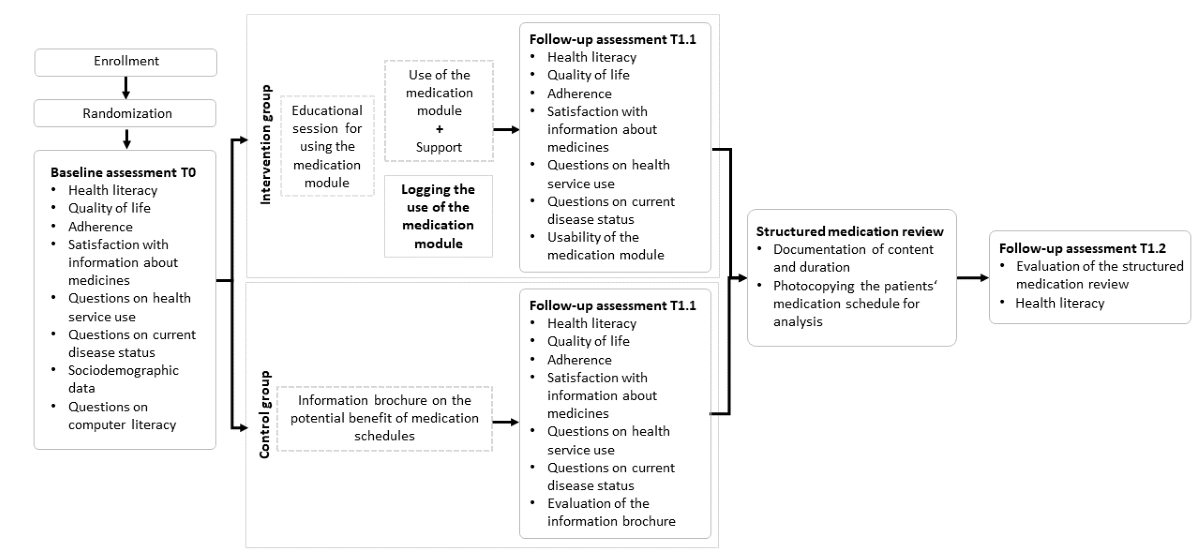
Study procedure of the prospective randomized controlled study. Data assessments at T0, T1.1, and T1.2 were done via questionnaires filled in by the patient during his or her visits to the primary care practice.

The study (see Figure 1) was conducted between May and October 2016. Each practice invited patients who met the inclusion criteria to participate in the study. Patients who agreed to participate were randomized by the medical assistant to the intervention group and the control group at a 1:1 ratio, following a previously created randomization list.

The study consisted of two patient visits in the primary care practice before (ie, baseline visit, T0) and after the intervention (ie, follow-up visit, T1). During these visits, there were three data-assessment points: one at T0 and two at T1, with T1.1 before the structured medication review and T1.2 after the medication review. During these time points, both the baseline information, such as sociodemographic information, and the patient- and process-oriented outcomes were documented using paper-based questionnaires, typically in the GP practice. All patient-oriented outcomes were measured using German versions of validated survey instruments.

During the baseline assessment, the patient filled in a questionnaire documenting health literacy using the Health Literacy Questionnaire (HLQ), which was developed by Osborne and colleagues [19,20]. Out of the eight subscales of the questionnaire, we selected the four subscales that were of particular interest for health literacy with regard to drug treatment: scales 5 (*appraisal of health information*), 6 (*ability to actively engage with health care providers*), 8 (*ability to find good health information*), and 9 (*understanding health information well enough to know what to do*). Moreover, patients filled in questionnaires regarding quality of life (ie, the World Health Organization Quality of Life instrument, brief version [WHOQOL-BREF]-global items [21]), self-reported adherence (ie, the Medication Adherence Report Scale, German version [MARS-D] [22]), satisfaction with drug information (ie, the Satisfaction with Information about Medicines Scale, German version [SIMS-D] [23,24]), and utilization of medical services (ie, the Mannheimer Modul Ressourcenverbrauch [MMRV] [25]). Patients also provided specified information regarding their sociodemographic and disease status (ie, blood pressure, hypoglycemia, hemoglobin A1c value, fasting blood glucose levels, and drug treatment) as well as current internet and computer use.

Directly after the baseline assessment, the patients in the control group received an information brochure on the potential benefit of medication schedules. In the intervention group, patients were scheduled for a training date to be educated about the use of the electronic medication module. The training session was performed by four study team members to ensure high reliability and consistent quality for all training sessions.

A total of 4-8 weeks after randomization, the medical assistant invited the patients to the primary care practice for a follow-up visit (T1). During this visit, patients received the same questionnaire that was administered during the baseline assessment, except that it contained only scales 5 and 8 from the HLQ and no sociodemographic data or questions about computer literacy. In the intervention group, the questionnaire additionally contained questions regarding the usability of the medication module (ie, the System Usability Scale [SUS] [26]), while in the control group, patients were asked about the comprehensibility of the information brochure. Subsequently, the medical assistant performed a structured medication review with each patient to document which drugs the patient was actually taking. During the medication review, potential problems with the actual medication were assessed and documented to be discussed later on with the GP [27]. If the patient possessed a printed medication schedule, the medical assistant would photocopy it but was instructed not to use this schedule during the medication review. The duration of the structured medication review was documented. After the structured medication review, both patient and medical assistant filled in a questionnaire evaluating the conduct of the medication review. The patient questionnaire additionally contained scales 6 and 9 from the HLQ. Throughout the intervention period, the use of the medication module (ie, number of log-ins and performed actions) was logged.

### Primary and Secondary Endpoints

As primary endpoint, the change in health literacy between T0 and T1 was assessed. As secondary endpoints, the following were evaluated: differences in quality of life, adherence, and satisfaction with drug information; differences in hemoglobin A1c, fasting blood glucose levels, and hypoglycemia; the course, time consumption, satisfaction, and evaluation of the structured medication review; the prevalence and quality of the patients’ medication schedules; and the use of, and satisfaction with, the medication module and the information brochure. This paper will focus on the general feasibility of the study design and will report the primary endpoint. Secondary endpoints will be aggregated and reported elsewhere.

### Statistical Analysis

We planned to enroll 120 patients (n=60 per group). This sample size was primarily based on matters of feasibility and allowed us to detect a standardized treatment effect of Cohen *d*=0.6 with a power of 1–beta=.9 when applying a two-sided *t* test with a two-sided significance level of alpha=.05.

We conducted two different types of analyses on two populations to assess primary and secondary outcomes. The intention-to-treat population comprised all randomized patients, while the per-protocol population comprised all randomized patients without protocol deviations. The primary endpoint was the difference between T1 and T0 in the sum score of scales 5, 6, 8, and 9 of the HLQ [19,20]. Each of the four scales is composed of five items with either four (scale 5) or five (scales 6, 8, and 9) Likert-scale values. Scale scores were determined by calculating the mean of all answered items, if at least three items per scale were answered. In case all four scale scores could be determined, the HLQ sum score was defined as the mean of all four sum scores and was otherwise set to missing.

Due to the hierarchical data structure, a multilevel analysis was conducted, with patients at level 1 and practice at level 2. The primary model was a linear mixed model with the HLQ score difference (T1–T0) as the dependent variable; treatment group and baseline HLQ score as fixed factor and covariate, respectively; and practice as a random factor, using the restricted maximum-likelihood method to fit the model. The primary analysis was conducted using the multilevel approach, assuming that missing values in the primary outcome can either be explained by the baseline HLQ score or the treatment group. Thus, no additional imputation of missing values was conducted.

Secondary endpoints such as scale scores were analyzed using the same multilevel approach. Effect estimates were calculated alongside 95% CIs and *P* values. Due to the exploratory character of the study, all *P* values were only of a descriptive nature, thus, no adjustment for multiple testing was performed. *P* values less than .05 were regarded as statistically significant. All analyses were conducted using SAS 9.4 (SAS Institute).

## Results

### Participants

Overall, 13 GPs agreed to participate and recruit patients, with one GP entrusting three medical assistants to coordinate the study and conduct the structured medication reviews. Hence, 15 medical assistants in 13 primary care practices were involved in randomization and data assessment. 

**Figure 2:**
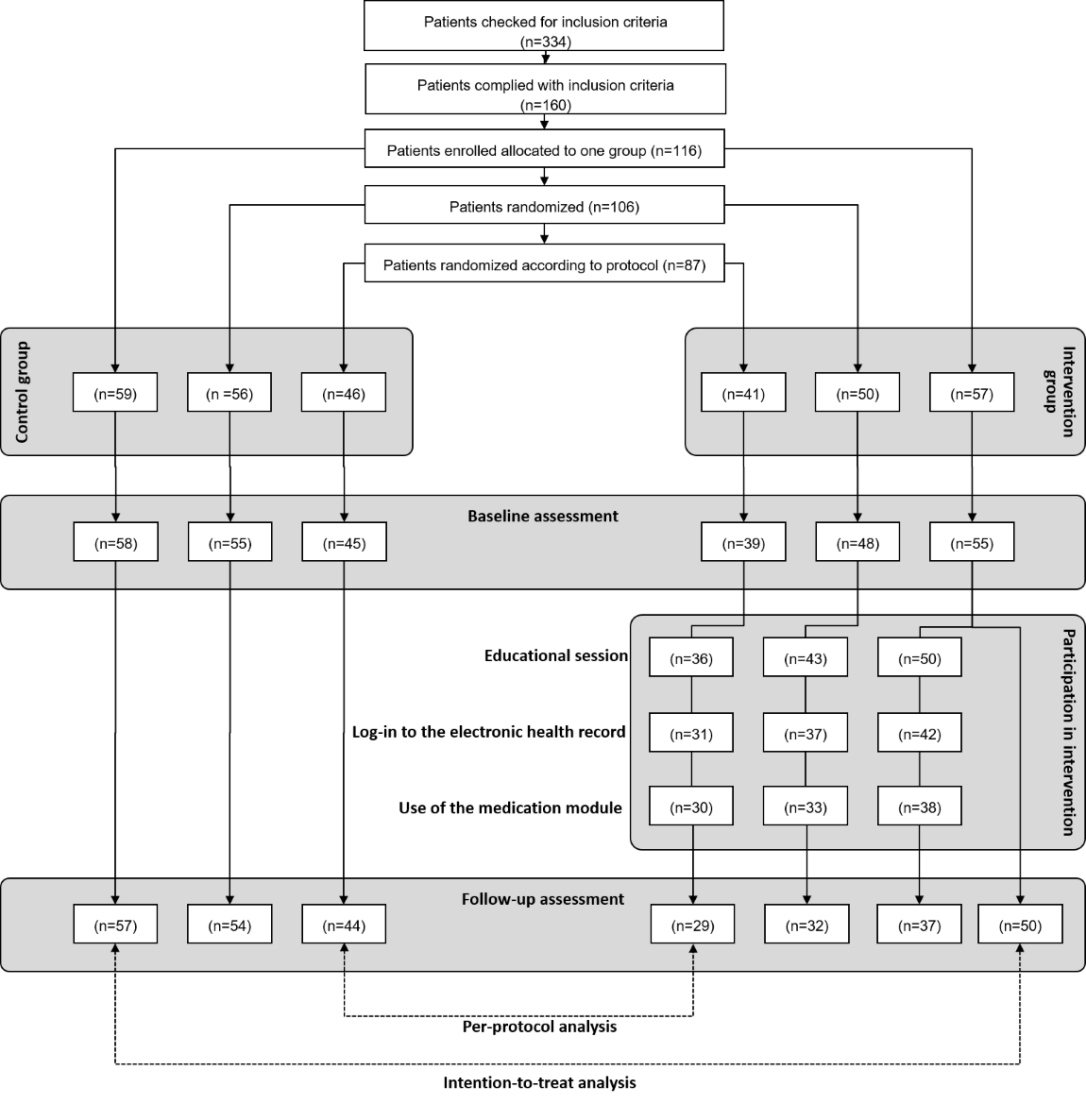
Patient flow during the study procedure. All patients from the intervention group are displayed on the right side of the flowchart and all patients from the control group are displayed on the left side. As not all patients were correctly enrolled in the study, there are three branches displayed on each side. Dropouts according to loss of follow-up are displayed vertically.

Overall, 116 patients agreed to participate in the study and were allocated to either the intervention or control group (see Figure 2). Of those, 113 (97.4%) participated in the baseline assessment and 107 (92.2%) also participated in the follow-up assessment. These participants were included in the intention-to-treat analysis. In total, 75.0% of included participants (87/116) were randomized according to the protocol. Of those, 73 participants (84%) followed the intervention as intended, completed the follow-up assessment, and were consequently eligible for per-protocol analysis. Table 1 presents the sociodemographic information as well as information about diagnosis and medication of all included participants.

**Table 1:** Characteristics of participants.

Characteristic		Intention-to-treat analysis			Per-protocol analysis		
		Intervention group (n=55)	Control group (n=58)	Total (n=113)	Intervention group (n=29)	Control group (n=44)	Total (n=73)
Age (years), mean (SD)		57.5 (11.2)	60.1 (10.2)	58.9 (10.8)	55.3 (10.5)	60.3 (10.8)	58.3 (10.9)
**Gender, n (%)**							
	Male	30 (56)	37 (64)	67 (59.8)	21 (75)	27 (61)	48 (67)
	Missing values	1 (2)	0 (0)	1 (0.9)	1 (4)	0 (0)	1 (1)
**Current employment, n (%)**							
	Employed	27 (50)	33 (57)	60 (53.6)	17 (61)	26 (59)	43 (60)
	Missing values	1 (2)	0 (0)	1 (0.9)	1 (4)	0 (0)	1 (1)
Living alone, n (%)		12 (22)	13 (22)	25 (22.1)	8 (28)	10 (23)	18 (25)
Comorbidities (yes), n (%)		50 (91)	48 (83)	98 (86.7)	27 (93)	37 (84)	64 (88)
**Diabetes diagnosis**							
	Time since diagnosis (years), mean (SD)	9.6 (8.8)	9.7 (7.4)	9.7 (8.1)	9.8 (7.9)	8.9 (7.4)	9.3 (7.6)
	Missing values, n (%)	6 (11)	11 (19)	17 (15.0)	4 (14)	10 (23)	14 (19)
**Drugs**							
	Number of drugs per day, mean (SD)	5.3 (3.3)	4.6 (2.7)	4.9 (3.0)	5.0 (3.1)	4.5 (3.0)	4.7 (3.0)
	Missing values, n (%)	3 (5)	3 (5)	6 (5.3)	1 (3)	3 (7)	4 (5)
**Diabetes medication, n (%)**							
	Tablets	34 (64)	40 (71)	74 (67.9)	17 (61)	29 (69)	46 (66)
	Tablets and injection	11 (21)	12 (21)	23 (21.1)	6 (21)	9 (21)	15 (21)
	Injection	8 (15)	3 (5)	11 (10.1)	5 (18)	3 (7)	8 (11)
	Other	0 (0)	1 (2)	1 (0.9)	0 (0)	1 (2)	1 (1)
	Missing values	2 (4)	2 (4)	4 (3.7)	1 (4)	2 (5)	3 (4)

### Health Literacy

Having access to the electronic medication platform did not have any significant effect on health literacy when comparing the two treatment groups, but it appeared to influence some aspects of health literacy when only assessing the effect in the intervention group (see Table 2). Results of the per-protocol analysis tended to be similar to those of the intention-to-treat analysis, albeit intervention group effects were slightly higher (see Table 3).

**Table 2:** Differences between Health Literacy Questionnaire (HLQ) sum scores and scores from scales 5, 6, 8, and 9 by group at baseline (T0) and follow-up visits (T1), including effect estimates by group adjusted for baseline value and two-level structure .

Measures		Intention-to-treat analysis, T1–T0 scores						Per-protocol analysis, T1–T0 scores					
		Intervention group		Control group		Total		Intervention group		Control group		Total	
**HLQ sum score (primary endpoint)**													
	Complete observations, n		50		55		105		29		43		72
	Mean (SD)		0.18 (0.34)		0.15 (0.39)		0.17 (0.37)		0.24 (0.36)		0.12 (0.34)		0.17 (0.35)
	Median		0.16		0.15		0.15		0.20		0.14		0.15
	Minimum, maximum		–0.35, 1.25		–1.35, 1.25		–1.35, 1.25		–0.31, 1.25		–1.35, 0.80		–1.35, 1.25
	Effect estimate		0.18		0.15				0.22		0.11		
	SE		0.05		0.05				0.07		0.06		
	*P* value		.001		.005				.002		.07		
	95% CI		0.07 to 0.28		0.05 to 0.25				0.09 to 0.36		–0.01 to 0.23		
**Scale 5: Appraisal of health information (four scale values)**													
	Complete observations, n		50		56		106		29		44		73
	Mean (SD)		0.11 (0.44)		0.16 (0.55)		0.13 (0.50)		0.09 (0.47)		0.08 (0.53)		0.09 (0.50)
	Median		0.00		0.20		0.00		0.00		0.00		0.00
	Minimum, maximum		–1.00, 1.60		–1.20, 1.80		–1.20, 1.80		–1.00, 1.60		–1.20, 1.80		–1.20, 1.80
	Effect estimate		0.16		0.11				0.12		0.06		
	SE		0.06		0.06				0.08		0.06		
	*P* value		.01		.05				.13		.32		
	95% CI		0.04 to 0.28		–0.00 to 0.23				–0.04 to 0.28		–0.06 to 0.19		
**Scale 6: Ability to actively engage with health care providers (five scale values)**													
	Complete observations, n		50		57		107		29		44		73
	Mean (SD)		0.86 (0.93)		0.93 (0.98)		0.90 (0.95)		0.99 (0.76)		0.88 (0.96)		0.92 (0.88)
	Median		0.88		0.80		0.80		1.00		0.70		0.80
	Minimum, maximum		–2.40, 3.00		–0.80, 3.20		–2.40, 3.20		–0.60, 3.00		–0.80, 3.20		–0.80, 3.20
	Effect estimate		0.81		0.95				0.95		0.86		
	SE		0.11		0.11				0.13		0.11		
	*P* value		<.001		<.001				<.001		<.001		
	95% CI		0.58 to 1.03		0.73 to 1.17				0.69 to 1.21		0.63 to 1.09		
**Scale 8: Ability to find good health information (five scale values)**													
	Complete observations, n		50		56		106		29		44		73
	Mean (SD)		–0.41 (0.70)		–0.36 (0.89)		–0.38 (0.81)		–0.30 (0.51)		–0.34 (0.84)		–0.33 (0.72)
	Median		–0.30		–0.20		–0.20		–0.20		–0.20		–0.20
	Minimum, maximum		–2.40, 1.00		–3.40, 2.20		–3.40, 2.20		–1.20, 1.00		–3.40, 1.20		–3.40, 1.20
	Effect estimate		–0.40		–0.41				–0.33		–0.39		
	SE		0.13		0.13				0.17		0.14		
	*P* value		.003		.002				.05		.009		
	95% CI		–0.66 to –0.14		–0.66 to –0.15				–0.66 to 0.00		–0.68 to –0.10		
**Scale 9: Understand health information well enough to know what to do (five scale values)**													
	Complete observations, n		50		56		106		29		43		72
	Mean (SD)		0.18 (0.62)		–0.06 (0.70)		0.05 (0.67)		0.18 (0.63)		–0.08 (0.68)		0.02 (0.67)
	Median		0.20		–0.20		0.00		0.20		0.00		0.00
	Minimum, maximum		–1.20, 2.00		–2.80, 1.60		–2.80, 2.00		–0.80, 2.00		–2.80, 1.40		–2.80, 2.00
	Effect estimate		0.12		–0.02				0.13		–0.09		
	SE		0.09		0.08				0.12		0.10		
	*P* value		.15		.83				.29		.39		
	95% CI		–0.05 to 0.30		–0.18 to 0.14				–0.11 to 0.37		–0.30 to 0.12		

**Table 3:** Intervention effects for the intervention group compared to the control group adjusted for baseline value and two-level structure: difference between baseline visit (T0) and follow-up visit (T1).

Scales		Effect^a^	SE	*P* value	95% CI	Cohen *d*^b^
**Intention-to-treat analysis**						
	Sum score	0.031	0.06	.62	–0.09 to 0.16	0.08
	Scale 5: Appraisal of health information	0.043	0.08	.60	–0.12 to 0.20	0.09
	Scale 6: Ability to actively engage with health care providers	–0.141	0.11	.21	–0.36 to 0.08	–0.15
	Scale 8: Ability to find good health information	0.004	0.14	.98	–0.27 to 0.28	0.01
	Scale 9: Understand health information well enough to know what to do	0.142	0.10	.17	–0.06 to 0.35	0.21
**Per-protocol analysis**						
	Sum score	0.116	0.08	.12	–0.03 to 0.27	0.33
	Scale 5: Appraisal of health information	0.058	0.10	.57	–0.14 to 0.26	0.12
	Scale 6: Ability to actively engage with health care providers	0.093	0.12	.44	–0.15 to 0.33	0.11
	Scale 8: Ability to find good health information	0.056	0.16	.73	–0.26 to 0.38	0.07
	Scale 9: Understand health information well enough to know what to do	0.219	0.13	.09	–0.04 to 0.48	0.33

^a^Treatment effect estimate: difference between the intervention group and control group. Positive effect estimates indicate an advantage of the intervention group over the control group.

^b^Standardized effect estimate adjusted for standard deviation.

**Figure 3:**
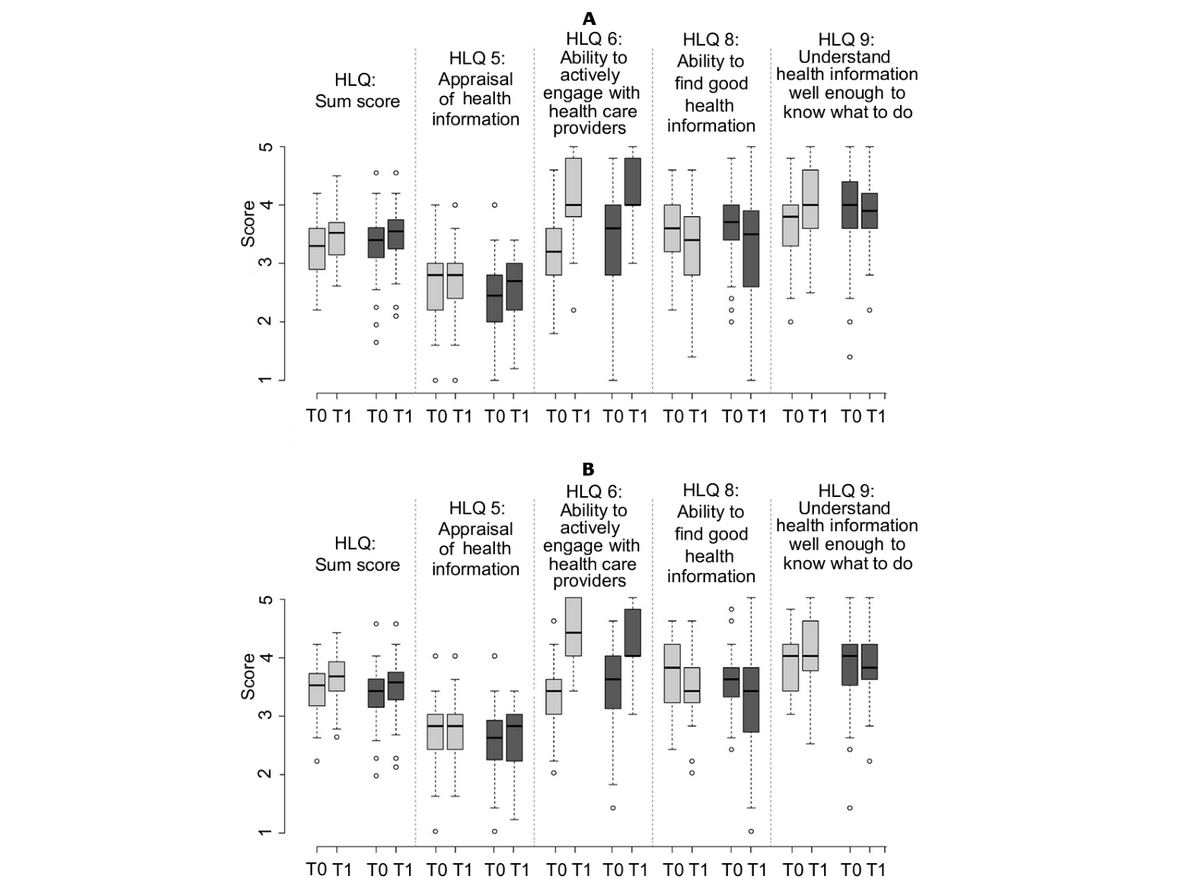
Results for health literacy. Graph A displays the results of the intention-to-treat analysis and Graph B displays the results of the per-protocol analysis. Patients from the intervention group are displayed in light gray and patients from the control group are displayed in dark gray. Health Literacy Questionnaire (HLQ) subscales are indicated by their numbers (5, 6, 8, and 9). T0: baseline visit; T1: follow-up visit.

Most included participants declared to have appraised the health information (HLQ scale 5) and to have been able to actively engage with health care providers (HLQ scale 6) similarly well both before and after taking part in the study (see Figure 3). Nevertheless, both the appraisal of health information and the ability to engage with health care providers were ranked slightly higher after participating, whereas the effect on the ability to engage with health care providers was greater and statistically significant (*P*<.001; see Table 2). However, this effect occurred in both groups (see Table 3).

Intervention and control groups differed with regard to HLQ scale 9, and participants of the intervention group were better able to understand health information well enough to know what to do than were the participants of the control group. The understanding slightly rose in the intervention group and slightly decreased in the control group at the follow-up assessment (see Figure 3), but the difference between treatment groups was not statistically significant (see Table 3). The ability to find good health information (HLQ scale 8) was estimated as slightly worse after participating than before (see Figure 3). Even here, no statistically significant difference between intervention and control groups could be observed (see Table 3).

## Discussion

### Principal Findings

In this controlled pilot study among T2DM patients, we assessed the impact of providing access to a PEPA with an interactive medication module on health literacy and found no change. This result should be critically discussed on several levels, as done in the following sections. This approach also highlights the limitations of the study.

### Selection of Assessment Method of Health Literacy

We assessed health literacy with a validated tool evaluating four of its key dimensions (see Tables 2 and 3). The instrument has been successfully used in previous studies and showed good efficacy in distinguishing different health literacy levels [19,20]. Moreover, it has been applied in intervention studies in a wide range of countries [28] and showed reliable results, while, at the same time, being shorter than other tools [28], suggesting that the questionnaire might be easier to use as an outcome measure tool. Indeed, the included patients dealt well with the provided questionnaires, as shown in the small number of missing values. The fact that we decided to use only four dimensions of the tool was owed to the fact that these dimensions explicitly focused on medication-related issues (ie, the main focus of our study). Obviously, we might have received different results if we had applied all eight dimensions or used other tools assessing health literacy, such as the Health Literacy Survey, German version (HLS-Ger) [29], which consists of 47 items and has shown a correlation between low health literacy and adherence among the German population. However, the following issues kept us from using this tool in our study: the length of the questionnaire, the fact that it assesses a broad concept of health literacy, and the fact that it was primarily developed to obtain and compare epidemiological data on health literacy in various populations, rather than to be used in intervention studies [30].

Hence, we believe that both the tool as well as the assessment method used in this study were appropriate and should have detected potential differences in health literacy, if they had occurred.

### Duration of the Intervention

Previous intervention studies that successfully addressed health literacy often had longer durations, typically lasting 9 months to 2 years [31-33]. Therefore, our study might have been too short to produce its full effects. This is also highlighted by the fact that not all patients in the intervention group actually accessed the PEPA.

### Health Literacy Level of Included Patients and Expected Effects

At the beginning of the study, our study participants already showed comparable or higher health literacy levels compared to previous cohorts that were included in intervention studies to improve health literacy, suggesting that ceiling effects might have occurred, making the detection of small changes more difficult.

A population-based Danish study assessing health literacy in subscales 6 and 9 of the HLQ among people with long-term chronic conditions revealed lower subscale means than in our study [34]. The results on the HLQ subscales in a study among an Australian population of older patients with multiple conditions showed higher mean values on *appraisal of health information* (mean 2.78) than in our study. The *ability to actively engage with health care professionals* was lower in our study compared to the Australian population (mean 3.97); however, this value increased and was higher after the intervention (mean 4.17), demonstrating the benefit of the contact with the medical assistants. The *ability to find good health information* showed lower mean values compared to the Australian population (mean 3.65), whereas in the subscale *understand health information enough to know what to do*, our values stayed within the 95% CI reported in the Australian population (95% CI 3.81-3.91) [35]. The comparability with this sample is restricted, as patients in our sample were younger; however, the results may indicate that the medication module was a support to patients regarding information finding and action.

Moreover, during the sample size calculation, we assumed that with the given sample size, only a relatively high standardized treatment effect of Cohen *d*=0.6 could be detected with a sufficiently high power. Since we only observed small-to-moderate effects, the highest being a Cohen *d* of 0.33 for the primary endpoint and scale 9 in the per-protocol population, our study sample size was too small to yield statistically significant treatment group differences but high enough to now thoroughly plan a prospective intervention study. Another potential reason for an insignificant study outcome might be that we did not enrich our study population with patients with low health literacy.

### Success of the Implementation

Strikingly high dropout rates among the intervention and control groups, which left only 73 instead of an initial 116 enrolled patients for per-protocol analysis, clearly suggest that insufficient power could be a reason for nonefficacy. However, the high dropout rates appeared in several stages of the study procedure; this is worth a detailed discussion to identify weaknesses and strengths of the study design and to derive lessons learned for future studies that can guide future complex interventions in this field.

After inclusion in the study, group allocation already appeared difficult for medical assistants, and of 116 patients, only 87 were randomized according to the protocol. Thereby, randomization according to the protocol either failed because patients were deliberately allocated to one group (n=10) or because the correct randomization scheme was not understood (n=19). Hence, incorrect randomization already diminished study power by about 10 percent-points as compared to the power that was originally aspired to of 90%, assuming a treatment effect of Cohen *d*=0.6.

Subsequently, dropouts occurred at the level of the baseline assessment, with one patient in the control group and two patients in the intervention group withdrawing consent or failing to keep their appointment for the baseline assessment. While these dropouts are typical for any type of intervention study, the dropout rate during the intervention phase, in particular, was unexpectedly large. This was either because the patients failed to attend the educational sessions where they were trained in the use of the medication platform or, even more often, because they never used the medication platform during the intervention phase. Indeed, after training, eight patients of the intervention group never even logged in to the PEPA and four more logged in at least once but never used the medication platform. Hence, almost one in three patients did not take part in the intervention, even though they agreed to take part in the study, filled in the baseline assessment, and participated in a training course. This high dropout rate during that stage was unexpected and certainly not considered during the sample size calculation.

Reasons for nonacceptance of the intervention were only qualitatively and sporadically assessed but included the fact that the intervention time was considered too short (eg, patients were on holidays during the entire intervention time) or that patients were not as comfortable in using computers as they had stated during the baseline screening.

Overall, the high dropout rates suggest that the study might have been underpowered. However, more importantly, it also highlights crucial pitfalls of health services interventions indicating that an intervention, as such, might be effective but must be carefully implemented and accepted by the people using it in order to result in effectiveness.

### Conclusions

The feasibility of this randomized controlled study was challenging, giving no indication whether the inadequate implementation or insufficient efficacy of the intervention, as such, contributed to the null effect of this study. This has important implications for the proper monitoring of the study quality and the many different steps of a complex intervention; this also stresses the need for meticulously planned and conducted pilot studies testing unrecognized sources of variability before pivotal intervention studies are implemented.

## Appendix

Multimedia Appendix 1 CONSORT‐EHEALTH checklist (V 1.6.1).

## Abbreviations

EHR: electronic health record
GP: general practitioner
HLQ: Health Literacy Questionnaire
HLS-Ger: Health Literacy Survey, German version
INFOPAT: INFOrmation Technology for PATient-oriented Healthcare in the Rhine-Neckar metropolitan region
MARS-D: Medication Adherence Report Scale, German version
MMRV: Mannheimer Modul Ressourcenverbrauch
P4/P5: Projects 4 and 5
PEPA: personal EHR
SIMS-D: Satisfaction with Information about Medicines Scale, German version
SUS: System Usability Scale
T0: baseline visit
T1: follow-up visit
T2DM: type 2 diabetes mellitus
WHOQOL-BREF: World Health Organization Quality of Life instrument, brief version
