# Supplementary material for: An Electronic Medication Module to Improve Health Literacy in Patients With Type 2 Diabetes Mellitus: Pilot Randomized Controlled Trial
Source: JMIR Form Res. 2020 Apr 28;4(4):e13746. doi: 10.2196/13746 (PMC7218604; doi:10.2196/13746)
Supplement: Multimedia Appendix 1 [file formative_v4i4e13746_app1.pdf]

CONSORT-EHEALTH Checklist V1.6.2 Report	Manuscript Number	13746
**(based on CONSORT-EHEALTH V1.6), available at [http://tinyurl.com/consort-ehealth-v1-6].**		
**Date completed**		
4/12/2020 18:06:51		
**by**		
Hanna Seidling		
An Electronic Medication Module To Improve Health Literacy in Patients With Type 2 Diabetes Mellitus: Randomized Controled Pilot Study		
TITLE		
**1a-i) Identify the mode of delivery in the title**		
"electronic medication module"....		
**1a-ii) Non-web-based components or important co-interventions in title**		
**1a-iii) Primary condition or target group in the title**		
... "patients with Type 2 Diabetes Mellitus"...		
ABSTRACT		
**1b-i) Key features/functionalities/components of the intervention and comparator in the METHODS section of the ABSTRACT**		
The Intervention and the comparator are explained in the Abstract (Methods section).		
**1b-ii) Level of human involvement in the METHODS section of the ABSTRACT**		
**1b-iii) Open vs. closed, web-based (self-assessment) vs. face-to-face assessments in the METHODS section of the ABSTRACT**		
**1b-iv) RESULTS section in abstract must contain use data**		
**1b-v) CONCLUSIONS/DISCUSSION in abstract for negative trials**		
INTRODUCTION		
**2a-i) Problem and the type of system/solution**		

"In 2012, a patient-controlled PEPA (personal EHR) was launched in the region of Rhine-Neckar in the southwest of Germany, within the INFOPAT (INFOrmation Technology for PATient-oriented Healthcare in the Rhine-Neckar metropolitan region) project [14]. The PEPA is "owned" by the patient so that he or she gains control over all disease-associated and medication-related data shared in the PEPA [15,16]. The PEPA offers the function of documentation but also actively involves patients in his or her process of care. In contrast to previous EHRs, patients can customize their PEPA themselves and give health care professionals access to its content [17]. To further support patients, the PEPA was equipped with an interactive medication module where patients can access information on different drugs, check drug-drug interactions, get evidence-based information on diabetes and its drug treatment, and compose and update their personal medication schedule in order to support medication self-management."		
**2a-ii) Scientific background, rationale: What is known about the (type of) system**		
"Patient portals are often interlinked with electronic health records (EHRs), which, until today, have the predominant aim to store all disease-related documentation, facilitating interchange and transfer of information between health care professionals. Current evidence on patients' attitudes toward patient portals suggest that patients might not use these portals on a regular basis [9], for instance, because they are not familiar with the functionalities they offer [10]. Even if the functionalities are known, the handling might still be complicated for the target population or they might be reluctant to use them, as has been shown for additional functionalities, such as secure messaging. Hence, these functionalities should always be assessed for potential barriers in usage [11]. However, if patients are individually informed and trained according to their needs on how to use the portal, and a particular effort is put on system usability from the beginning, patient portals might be useful tools to strengthen the patient's role [10,12,13]."		
**Does your paper address CONSORT subitem 2b?**		
"The aim of this study was to pilot the feasibility of the study procedure (ie, patient recruitment, randomization, data assessment, and documentation in the primary care practices) and of the intervention (ie, conduction of training episodes and accessibility of the medication module), while at the same time assessing the influence of the medication module on health literacy."		
METHODS		
**3a) CONSORT: Description of trial design (such as parallel, factorial) including allocation ratio**		

"We performed an exploratory, prospective, randomized controlled study with two data collection points among community-dwelling patients attending primary care practices."		
**3b) CONSORT: Important changes to methods after trial commencement (such as eligibility criteria), with reasons**		
There were no changes to Methods after trial commencement		
**3b-i) Bug fixes, Downtimes, Content Changes**		
**4a) CONSORT: Eligibility criteria for participants**		
"Patients were eligible if they were 18 years of age or older, were diagnosed with T2DM and treated with oral antidiabetic drugs and/or insulin, were considered mentally and physically capable of participating in the study, were generally familiar with computer use, had access to a computer or mobile device with internet access and an email account, and consented in written form to participate in the study. Patients not fulfilling the inclusion criteria could not participate in the study; apart from that, there were no formal exclusion criteria"		
**4a-i) Computer / Internet literacy**		
**4a-ii) Open vs. closed, web-based vs. face-to-face assessments:**		
This Information is given throughout the manuscript whenever appropriate		
**4a-iii) Information giving during recruitment**		
**4b) CONSORT: Settings and locations where the data were collected**		
Information is given throughout the manuscript - Data were collected in the GP practices, practices were located in the state of Baden-Württemberg		
**4b-i) Report if outcomes were (self-)assessed through online questionnaires**		
"During these time points, both the baseline information, such as sociodemographic information, and the patient- and process-oriented outcomes were documented using paper-based questionnaires, typically in the GP practice"		
**4b-ii) Report how institutional affiliations are displayed**		
**5) CONSORT: Describe the interventions for each group with sufficient details to allow replication, including how and when they were actually administered**		
**5-i) Mention names, credential, affiliations of the developers, sponsors, and owners**		
**5-ii) Describe the history/development process**		
**5-iii) Revisions and updating**		
**5-iv) Quality assurance methods**		
**5-v) Ensure replicability by publishing the source code, and/or providing screenshots/screen-capture video, and/or providing flowcharts of the algorithms used**		

**5-vi) Digital preservation**		
**5-vii) Access**		
initial contact: "In the intervention group, patients<br>were scheduled for a training date to be educated about the use<br>of the electronic medication module. The training session was<br>performed by four study team members to ensure high reliability<br>and consistent quality for all training sessions."<br>Subsequently, the patients had Access to the platform for About 8 weeks until T1 assessment. They could enter the platform whenever they wnated		
**5-viii) Mode of delivery, features/functionalities/components of the intervention and comparator, and the theoretical framework**		
"Finally, the module consisted<br>of a documentation submodule for the patients' medication<br>regimen and a general information portal on drug administration.<br>The documentation submodule facilitated searching all drugs available on the German drug market, putting together a<br>medication regimen, and entering additional information, such<br>as drug dosage, indication, and administration advice.<br>Subsequently, the patients could store the medication regimen<br>to access and modify in the future. Moreover, they could print<br>out a medication schedule containing all previously entered<br>information. For each drug, the patient had access to basic drug<br>information, such as the package information leaflet and more<br>detailed information on drug administration (eg, on whether a<br>tablet could be split or crushed). Moreover, the entire medication<br>regimen was checked for drug-drug interactions involving<br>over-the-counter drugs as well as contraindicated combinations"<br>There was no particular theoretical Framework for the developement of the medication module.		
**5-ix) Describe use parameters**		
**5-x) Clarify the level of human involvement**		
**5-xi) Report any prompts/reminders used**		
There were no reminders. This implicetly results from the description of the Intervention.		
**5-xii) Describe any co-interventions (incl. training/support)**		
"Before using the medication module for the first time, each<br>patient completed a 45-minute, face-to-face, hands-on structured<br>training session by a member of the study team and printed<br>education material on how to use the module. Moreover, patients<br>could approach a first-level support person in case they had<br>problems using the medication module of the PEPA."		
**6a) CONSORT: Completely defined pre-specified primary and secondary outcome measures, including how and when they were assessed**		

"As primary endpoint, the change in health literacy between T0 and T1 was assessed. As secondary endpoints, the following were evaluated: differences in quality of life, adherence, and satisfaction with drug information; differences in hemoglobin A1c, fasting blood glucose levels, and hypoglycemia; the course, time consumption, satisfaction, and evaluation of the structured medication review; the prevalence and quality of the patients' medication schedules; and the use of, and satisfaction with, the medication module and the information brochure."<br><br>"The study consisted of two patient visits in the primary care practice before (ie, baseline visit, T0) and after the intervention (ie, follow-up visit, T1). During these visits, there were three data-assessment points: one at T0 and two at T1, with T1.1 before the structured medication review and T1.2 after the medication review. During these time points, both the baseline information, such as sociodemographic information, and the patient- and process-oriented outcomes were documented using paper-based questionnaires. All patient-oriented outcomes were measured using German versions of validated survey instruments."		
**6a-i) Online questionnaires: describe if they were validated for online use and apply CHERRIES items to describe how the questionnaires were designed/deployed**		
**6a-ii) Describe whether and how "use" (including intensity of use/dosage) was defined/measured/monitored**		
**6a-iii) Describe whether, how, and when qualitative feedback from participants was obtained**		
**6b) CONSORT: Any changes to trial outcomes after the trial commenced, with reasons**		
Information is given throughout the manuscript - Data were collected in the GP practices, practices were located in the state of Baden-Württemberg		
**7a) CONSORT: How sample size was determined**		
**7a-i) Describe whether and how expected attrition was taken into account when calculating the sample size**		
**7b) CONSORT: When applicable, explanation of any interim analyses and stopping guidelines**		

"As primary endpoint, the change in health literacy between T0 and T1 was assessed. As secondary endpoints, the following were evaluated: differences in quality of life, adherence, and satisfaction with drug information; differences in hemoglobin A1c, fasting blood glucose levels, and hypoglycemia; the course, time consumption, satisfaction, and evaluation of the structured medication review; the prevalence and quality of the patients' medication schedules; and the use of, and satisfaction with, the medication module and the information brochure."<br><br>"The study consisted of two patient visits in the primary care practice before (ie, baseline visit, T0) and after the intervention (ie, follow-up visit, T1). During these visits, there were three data-assessment points: one at T0 and two at T1, with T1.1 before the structured medication review and T1.2 after the medication review. During these time points, both the baseline information, such as sociodemographic information, and the patient- and process-oriented outcomes were documented using paper-based questionnaires. All patient-oriented outcomes were measured using German versions of validated survey instruments."		
**8a) CONSORT: Method used to generate the random allocation sequence**		
Only patients were randomized.		
**8b) CONSORT: Type of randomisation; details of any restriction (such as blocking and block size)**		
Randomization was done on Patient Level, stratefied by GP practice.<br><br>"Patients who agreed<br>to participate were randomized by the medical assistant to the intervention group and the control group at a 1:1 ratio, following a previously created randomization list."		
**9) CONSORT: Mechanism used to implement the random allocation sequence (such as sequentially numbered containers), describing any steps taken to conceal the sequence until interventions were assigned**		
n/a		
**10) CONSORT: Who generated the random allocation sequence, who enrolled participants, and who assigned participants to interventions**		
Described throughout the manuscript.<br>Randomization lists were provided by the study Team, Patient recruitement and randomization was done in the GP practices.		
**11a) CONSORT: Blinding - If done, who was blinded after assignment to interventions (for example, participants, care providers, those assessing outcomes) and how**		
**11a-i) Specify who was blinded, and who wasn't**		
nobody was blinded.		
**11a-ii) Discuss e.g., whether participants knew which intervention was the "intervention of interest" and which one was the "comparator"**		
**11b) CONSORT: If relevant, description of the similarity of interventions**		
n/a		
**12a) CONSORT: Statistical methods used to compare groups for primary and secondary outcomes**		

see section on statistical Analysis:<br>"We conducted two different types of analyses on two populations to assess primary and secondary outcomes. The intention-to-treat population comprised all randomized patients, while the per-protocol population comprised all randomized patients without protocol deviations. The primary endpoint was the difference between T1 and T0 in the sum score of scales 5, 6, 8, and 9 of the HLQ [19,20]. Each of the four scales is composed of five items with either four (scale 5) or five (scales 6, 8, and 9) Likert-scale values. Scale scores were determined by calculating the mean of all answered items, if at least three items per scale were answered. In case all four scale scores could be determined, the HLQ sum score was defined as the mean of all four sum scores and was otherwise set to missing. Due to the hierarchical data structure, a multilevel analysis was conducted, with patients at level 1 and practice at level 2. The primary model was a linear mixed model with the HLQ score difference (T1–T0) as the dependent variable; treatment group and baseline HLQ score as fixed factor and covariate, respectively; and practice as a random factor, using the restricted maximum-likelihood method to fit the model. The primary analysis was conducted using the multilevel approach, assuming that missing values in the primary outcome can either be explained by the baseline HLQ score or the treatment group. Thus, no additional imputation of missing values was conducted."		
**12a-i) Imputation techniques to deal with attrition / missing values**		
see section on statistical Analysis (already copy pasted above)		
**12b) CONSORT: Methods for additional analyses, such as subgroup analyses and adjusted analyses**		
We did not do subgroup Analysis, however we performed a ITT and a PP Analysis as described.		
RESULTS		
**13a) CONSORT: For each group, the numbers of participants who were randomly assigned, received intended treatment, and were analysed for the primary outcome**		
See table 2 and 3 as well as figure 3.		
**13b) CONSORT: For each group, losses and exclusions after randomisation, together with reasons**		
see figure 2 as well as result section and discussion		
**13b-i) Attrition diagram**		
see figure 2		
**14a) CONSORT: Dates defining the periods of recruitment and follow-up**		
see Methods section (study Duration and time between T0 and T1)		
**14a-i) Indicate if critical “secular events” fell into the study period**		
**14b) CONSORT: Why the trial ended or was stopped (early)**		
n/a		
**15) CONSORT: A table showing baseline demographic and clinical characteristics for each group**		
see table 1		

**15-i) Report demographics associated with digital divide issues**		
see table 1		
**16a) CONSORT: For each group, number of participants (denominator) included in each analysis and whether the analysis was by original assigned groups**		
**16-i) Report multiple "denominators" and provide definitions**		
see respective tables and text		
**16-ii) Primary analysis should be intent-to-treat**		
**17a) CONSORT: For each primary and secondary outcome, results for each group, and the estimated effect size and its precision (such as 95% confidence interval)**		
in this paper only the Primary endpoint is reported (see Methods section)		
**17a-i) Presentation of process outcomes such as metrics of use and intensity of use**		
**17b) CONSORT: For binary outcomes, presentation of both absolute and relative effect sizes is recommended**		
see tables and text		
**18) CONSORT: Results of any other analyses performed, including subgroup analyses and adjusted analyses, distinguishing pre-specified from exploratory**		
all Analyses for the Primary endpoint are presented		
**18-i) Subgroup analysis of comparing only users**		
**19) CONSORT: All important harms or unintended effects in each group**		
we did not particularly assess harm		
**19-i) Include privacy breaches, technical problems**		
**19-ii) Include qualitative feedback from participants or observations from staff/researchers**		
DISCUSSION		
**20) CONSORT: Trial limitations, addressing sources of potential bias, imprecision, multiplicity of analyses**		
**20-i) Typical limitations in ehealth trials**		
the entire discussion basically deals with potential shortcomings and limitations of the study		
**21) CONSORT: Generalisability (external validity, applicability) of the trial findings**		
**21-i) Generalizability to other populations**		
**21-ii) Discuss if there were elements in the RCT that would be different in a routine application setting**		
**22) CONSORT: Interpretation consistent with results, balancing benefits and harms, and considering other relevant evidence**		
**22-i) Restate study questions and summarize the answers suggested by the data, starting with primary outcomes and process outcomes (use)**		
the discussion section basically goes through all potential limitations of the study		
**22-ii) Highlight unanswered new questions, suggest future research**		
Other information		
**23) CONSORT: Registration number and name of trial registry**		

No, the study wasn`t registered		
**24) CONSORT: Where the full trial protocol can be accessed, if available**		
No, the study protocol isn`t accessible		
**25) CONSORT: Sources of funding and other support (such as supply of drugs), role of funders**		
"The study was conducted within the INFOPAT project [14], which was supported by grants from the German Federal Ministry of Education and Research (funding code 01KQ1003B)"		
**X26-i) Comment on ethics committee approval**		
**x26-ii) Outline informed consent procedures**		
**X26-iii) Safety and security procedures**		
**X27-i) State the relation of the study team towards the system being evaluated**		
